# The Epidemics of the Middle Ages

**Published:** 1844-07-01

**Authors:** 


THE EPIDEMICS OF THE MIDDLE AGES. From the German of *J. F. C. Hecker*, M.D. Translated by *B. G. Babington*, M.D. F.R.S., &c. Octavo, pp. 418. London, 1844.

### I. THE BLACK DEATH.

THE volume before us is the first publication of the Sydenham Society, and contains three translations from the celebrated Hecker, viz. the "BLACK DEATH"—the "DANCING MANIA"—and the "SWEATING SICKNESS," of the Middle Ages. Those dreadful scourges that have, at longer or shorter intervals, desolated the earth and thinned the ranks of mankind, must always prove highly interesting to the physician and the philosopher; but we are far less sanguine than either Hecker or his Translator, that the most accurate and authentic history of these terrific visitations will ever throw light on their nature and cause—perhaps not even on their treatment. Thus, what do we know of the origin, cause, or nature of cholera, which we have all witnessed a few years ago, more than we knew before the advent of the pestilence? Has the dire experience of that epidemic led us to any successful mode of treatment? It would not be easy to answer these questions in the affirmative. The absurd notions that have prevailed in all countries and in all visitations of this kind, that the wells were poisoned, sprung up, even in this enlightened age, when cholera raged throughout the land! True, there were not so many barbarous sacrifices of human life, at the altars of ignorance and prejudice, as in the days of Thucydides, or in the times of the "Black Death;" but still the ridiculous suspicion was harboured by thousands in this country and on the Continent, little more than ten years ago! The talented translator himself seems aware of the mysterious nature of these fearful scourges, and the difficulty of accounting for them on any theory hitherto broached.

"The contagionist and the anti-contagionist may each find ample support for his belief in particular cases; but in the construction of a theory sufficiently comprehensive to explain throughout, the origin and dissemination of universal disease, we shall not only perceive the insufficiency of either doctrine, taken singly, but after admitting the combined influence of both, shall even then find our views too narrow, and be compelled, in our endeavours to explain the facts, to acknowledge the existence of *unknown powers*, wholly unconnected either with communication by contact or atmospheric contamination."—*Preface*, xxiv.

We were somewhat amused with our Translator's apology for delaying the publication "till a moment when it is presumed that men's thoughts will be especially directed to the approaching hour of public thanksgiving." Alas! good, pious, and most religious Doctor, if you allude to Christmas or Easter, you are most egregiously mistaken! The elders of your readers will be far more occupied with the thoughts of roast beef, plum-pudding, and "red Port," than with the "Black Death" of the Middle Ages:—while the younger ones will think a great deal more of balls, concerts, quadrilles, and waltzes, than of the "Dancing Mania" of the Fourteenth

Century! If it be Easter that is alluded to, the steamings to and from Greenwich will leave little time for reflection on the "Sweating Sickness" of our forefathers. But the Translator is too modest. His work will survive many a Christmas and Easter, and be read long after the present generation are in their graves.

### THE DISEASE.

The Black Death appears to have been a real Oriental plague, presenting many of the essential characters exhibited by that malady down to the present day. It desolated Asia, Africa, and Europe in the Fourteenth Century, and swept away a fourth of the population of the Old World in four years, while in England the rate of mortality was double that! The disease was characterised by inflammatory boils and tumors of the glands—black spots on the skin—affections of the head—black tongue and fauces—burning thirst, &c. These were the ordinary symptoms, though not always present in the same case. But there were others of a graver dye. The organs of respiration were seized with a putrid inflammation, with violent pain in the chest, while blood of a pestiferous odour was expectorated from the lungs. In the West of Europe the predominating phenomena were, ardent fever, with evacuation of blood, often proving fatal in three days. Carbuncular affections of the lungs frequently destroyed life before buboes had time to come out. It was believed that the effluvia of the breath spread the disease far and near. At Florence it commenced with bleeding at the nose—a most dangerous symptom—then followed buboes and tumors indiscriminately all over the body, with black or blue splotches on the arms, thighs, &c. No medicine appeared to be of any use, and death was generally within three days.

"In England the malady appeared, as at Avignon, with spitting of blood, and with the same fatality, so that the sick who were afflicted either with this symptom or with vomiting of blood, died in some cases immediately, in others within twelve hours, or at the latest, in two days. The inflammatory boils and buboes in the groins and axillæ were recognised at once as prognosticating a fatal issue, and those were past all hope of recovery in whom they arose in numbers all over the body. It was not till towards the close of the plague that they ventured to open, by incision, these hard and dry boils, when matter flowed from them in small quantity, and thus, by compelling nature to a critical suppuration, many patients were saved. Every spot which the sick had touched, their breath, their clothes, spread the contagion; and, as in all other places, the attendants and friends who were either blind to their danger or heroically despised it, fell a sacrifice to their sympathy. *Even the eyes of the patient were considered as sources of contagion, which had the power of acting at a distance, whether on account of their unwonted lustre or the distortion which they always suffer in plague, or whether in conformity with an ancient notion, according to which the sight was considered as the bearer of a demoniacal enchantment.*" 7.

Doubtless the Mesmerists will press the "clair-voyance" of the plague patients into their service. The Chronicles report that only one-tenth of the inhabitants of England remained alive!!

The various histories of the various parts of Europe and Asia where the "Black Death" appeared, leave no doubt that the malady exhibited all the characteristic features of the modern plague. It is hardly necessary to say that the contagious nature of the disease was firmly believed by the physicians and people of the Fourteenth Century, as well as by Hecker himself, and the great majority of medical practitioners of the present day.

### CAUSES AND DIFFUSION.

It appears, from the researches of Hecker and others, that " great revolutions in the organism of the earth," had not long preceded the eruptions of the Epidemics of the Middle Ages. In China, parching droughts were followed by frightful storms and inundations—more than 400,000 people being drowned in the floods. Mountains fell in, and clefts were opened into the bowels of the earth; after which a pestilence rose and carried off five millions of people! In Europe, various terrestrial commotions accompanied or succeeded those of Asia. In the Island of Cyprus, earthquakes, hurricanes, and inundations ushered in the plague of the East, by which this blooming isle was converted into a desert. The atmosphere, which preserves such a remarkable uniformity in all places and in all seasons, evinced very unusual phenomena in the Fourteenth Century. Stinking fogs and mists spread from East to West—while pestilent vapours issued from the deep rents in the earth, and exhaled from the vast marshes caused by the inundations. These circumstances may, in some degree, account for the grave and frequent affections of the respiratory organs. The earthquakes were destructive, and almost universal, while strange meteors appeared in the air and terrified whole nations. The order of the seasons seemed to be inverted—crops failed—and famine added its sufferings to pestilence.

" In the progress of connected natural phenomena, from East to West, that great law of nature is plainly revealed which has so often and evidently manifested itself in the earth's organism, as well as in the state of nations dependent upon it. In the inmost depths of the globe, that impulse was given in the year 1333, which in uninterrupted succession for six-and-twenty years shook the surface of the earth, even to the western shores of Europe. From the very beginning the air partook of the terrestrial concussion, atmospherical waters overflowed the land, or its plants and animals perished under the scorching heat. The insect tribe was wonderfully called into life, as if animated beings were destined to complete the destruction which astral and telluric powers had begun. Thus did this dreadful work of nature advance from year to year; it was a progressive infection of the Zones, which exerted a powerful influence both above and beneath the surface of the earth; and after having been perceptible in slighter indications, at the commencement of the terrestrial commotions in China, convulsed the whole earth." 18.

M. Hecker considers the BLACK DEATH to have been contagious and infectious, in the highest degree, while the constitution of earth and air rendered it universally epidemic.

### MORTALITY.

We have no certain measure, like modern statistics, to estimate the ravages of the black plague. The probabilities were, according to Hecker, that, in Kairo, from ten to fifteen thousand died *daily*. In China, more than thirteen millions were swept away—India was depopulated—Tartary, Mesopotamia, Syria, and Armenia were covered with dead bodies—in

Caramania and Cæsarea, none were left alive! It would be needless to pursue the conjectural enumeration of deaths from this frightful scourge. The mortality must have been greatly beyond all idea which we can form from those epidemics that have happened in modern times.

It appears that more accurate records of the mortality were kept in England than in any other country. In Yarmouth 7052 died. In London there was one burial-ground, where fifty thousand bodies were deposited, arranged in layers. Throughout the country scarcely a tenth part remained alive! It is wonderful that Europe could have so soon got over such a dreadful shock and destruction of its population. It was universally observed that, after this dire calamity, women became greatly more prolific, and population increased in a far higher ratio than before.

MORAL EFFECTS.

These were various—some good, some evil. The pious closed their accounts with the world—their only remaining desire being the consolations of religion. Repentance seized the wicked—they resolved to forsake their vices and atone for their crimes, before being summoned hence. But hypocrisy, illusion and bigotry also stalked abroad. The *Flagellant* brotherhood started into existence to preach to the people, and offer up prayers for protection from the plague. These became numerous, and formidable to the Church. Then the suspicion arose that the Jews were at the bottom of the pestilence, and poisoned the people by means of pest-powders and other diableries! The rack soon caused numbers of the unhappy chosen people to confess murders and atrocities of which they were perfectly guiltless!

"When the evil had become universal," (speaking of Florence,) "the hearts of all the inhabitants were closed to feelings of humanity. They fled from the sick and all that belonged to them, hoping by these means to save themselves. Others shut themselves up in their houses, with their wives, their children and households, living on the most costly food, but carefully avoiding all excess. None were allowed access to them; no intelligence of death or sickness was permitted to reach their ears; and they spent their time in singing and music, and other pastimes. Others, on the contrary, considered eating and drinking to excess, amusements of all descriptions, the indulgence of every gratification, and an indifference to what was passing around them, as the best medicine, and acted accordingly. They wandered day and night, from one tavern to another, and feasted without moderation or bounds. In this way they endeavoured to avoid all contact with the sick, and abandoned their houses and property to chance, like men whose death-knell had already tolled." 47.

Such is the inconsistency, the instability—the utter worthlessness of the human mind!

PHYSIC AND PHYSICIANS.

The medical faculty of Paris was called upon to deliver its opinion on the causes of the "BLACK PLAGUE," and furnish appropriate therapeutic indications. A passage from their etiology will furnish some amusement to our readers.

"It is known that in India, and the vicinity of the Great Sea, the constellations which combated the rays of the sun, and the warmth of the heavenly fire, exerted their power especially against that sea, and struggled violently with its waters. (Hence, vapours often originate which envelope the sun, and convert his light into darkness.) These vapours alternately rose and fell for twenty-eight days; but at last, sun and fire acted so powerfully upon the sea, that they attracted a great portion of it to themselves, and the waters of the ocean arose in the form of vapour; thereby the waters were, in some parts, so corrupted, that the fish which they contained, died. These corrupted waters, however, the heat of the sun could not consume, neither could other wholesome water, hail or snow, and dew, originate therefrom. On the contrary, this vapour spread itself through the air in many places on the earth, and enveloped them in fog." 51.

So much for the ETIOLOGY of the BLACK DEATH, as propounded by the College of Physicians, 1346. We can only make room for a very short extract from the hygienic rules laid down by the said learned College.

"We are of opinion, that the constellations, with the aid of Nature, strive, by virtue of their divine might, to protect and heal the human race; and to this end, in union with the rays of the sun, acting through the power of fire, endeavour to break through the mist. Accordingly, within the next ten days, and until the 17th of the ensuing month of July, this mist will be converted into a stinking deleterious rain, whereby the air will be much purified. Now, as soon as this rain shall announce itself, by thunder or hail, every one of you should protect himself from the air; and, as well before as after the rain, kindle a large fire of vine-wood, green laurel, or other green wood; wormwood and chamomile should also be burnt in great quantity in the market-places, in other densely inhabited localities, and in the houses. Until the earth is again completely dry, and for three days afterwards, no one ought to go abroad in the fields." 52.

This reminds us of the sage rules and cautions issued from a high source in our own times, when the cholera was approaching our shores. We all remember how strenuously we were warned to have no intercourse with the world, if we could possibly avoid it—and, if unavoidable, to let down buckets from our attick windows, to haul up provisions, which were to be well fumigated with chloride of lime, before we attempted to swallow them!! We need not, therefore, indulge in deep laughter at the instructions of the Paris Faculty four hundred years ago.

There were several physicians, however, at that remote period, who rose far superior to the collective wisdom of the Parisian College. Guy de Chauliac was a star of considerable magnitude in those melancholy times. A conjunction of Saturn, Jupiter, and Mars in the sign of Aquarius, was considered by him and the other medical magnates, as the evident cause of the pestilence, and a corruption of the blood, as the immediate effect, or proximate cause of the epidemy. Bleeding and purging were the prominent measures employed, together with a host of ridiculous and unimportant remedies which need not now be noticed. Segregation was a prominent feature in the treatment, or rather the prevention of the malady, and our quarantines still keep up the charter, to the great annoyance of commerce, and without a particle of utility. It is a precious remnant of the wisdom of our ancestors—of which the following specimen deserves record. The first regulation is dated 17 January, 1374, and enjoins that "every plague-patient is to be taken out of the city (Reggio) into the fields, there to die or recover." The priests were to examine suspected persons and report them under punishment of being *burnt alive!* Thus ends our sketch of the "BLACK DEATH." The record of its devastations is melancholy and curious; but we do not see that it throws one single ray of light on the etiology, pathology, or therapœia of the direful epidemic.

### II. The Dancing Mania.

The Black Death had scarcely closed the graves over millions of the human race, when a strange superstition arose in that cradle of ideality—Germany, which, in the quaint language of Hecker, " hurried away body and soul into the magic circle of hellish superstition." It was termed the Chorea St. Viti, on account of the Bacchanalian leaps which characterized it, causing those affected to scream and foam, as though they were possessed by Satan. It seemed to be propagated over all Germany, and beyond its bounds, by sight or imitation. Early in 1374, assemblages of men and women were seen at Aix-la-Chapelle, forming themselves into circles, hand in hand, dancing for hours together, in wild delirium, till they fell to the ground in a state of exhaustion, groaning as if in the agonies of death. After a time, they recovered till another attack took place. Their abdomens became tympanitic in their paroxysms, and tight swathing relieved them after the accession. During the dance they neither saw nor heard, but were haunted by visions, and wild fancies. In many, an epileptic paroxysm preceded the dancing fit.

" In Liege, Utretcht, Tongres, and many other towns of Belgium, the dancers appeared with garlands in their hair, and their waists girt with cloths, that they might, as soon as the paroxysm was over, receive immediate relief on the attack of the tympany. This bandage was, by the insertion of a stick, easily twisted tight: many, however, obtained more relief from kicks and blows, which they found numbers of persons ready to administer; for, wherever the dancers appeared, the people assembled in crowds to gratify their curiosity with the frightful spectacle." 89.

The evil increased and spread to an alarming state, and the priests endeavoured to exorcise the dæmon that possessed the people, who rose upon the clergy and threatened them with destruction. Dr. Hecker describes over and over again, with most needless minuteness, the phenomena of this dancing mania, as it appeared in different places, and then he endeavours to trace its causes, but with little success. He thinks they were in some degree connected with certain ancient festivals in honour of St. John the Baptist. Paracelsus was the physician who distinguished himself most in the management of the dancing mania. A sample of his therapeutics will be sufficient.

" For the second kind of St. Vitus's dance, arising from sensual irritation, with which women were far more frequently affected than men, Paracelsus recommended harsh treatment and strict fasting. He directed that the patients should be deprived of their liberty; placed in solitary confinement, and made to sit in an uncomfortable place, until their misery brought them to their senses and to a feeling of penitence. He then permitted them gradually to return to their accustomed habits. Severe corporal chastisement was not omitted; but, on the other hand, angry resistance on the part of the patient was to be sedulously avoided, on the ground that it might increase his malady, or even destroy him: moreover, where it seemed proper, Paracelsus allayed the excitement of the nerves by immersion in cold water. On the third kind we shall not here enlarge. It was to be effected by all sorts of wonderful remedies, composed of the quintessences; and it would require, to render it intelligible, a more extended exposition of peculiar principles than suits our present purpose." 103.

With the Sixteenth Century the dancing mania seems to have expired, and was converted into the fighting mania, which raged during a great part of the Seventeenth Century, with still more fury and fatality than its predecessor.

We shall not follow our author through the details of this mania, as it appeared in Belgium, Germany, Italy, Abyssinia and other parts of the globe, modified, of course, by climate, manners, religion, &c. The Black Death or the Sweating-Sickness may possibly return; but St. Vitus, except in sporadic cases, is not very likely to pay an epidemic visit to Europe again.

### III. THE SWEATING SICKNESS.

This forms the third and concluding Article in the first volume of the Sydenham Society. To Englishmen it is more interesting than either of its predecessors—the "BLACK DEATH," or the "DANCING MANIA." This dreadful epidemic broke out in 1485, soon after the battle of Bosworth. It was a violent inflammatory fever, ushered in by a rigor, and succeeded by a most remarkable prostration—oppression at the præcordia—headache—stupor, and profuse fetid perspiration. The Crisis, favourable or otherwise, was over in the space of a day and a night. The internal heat was insufferable; and yet refrigerants were fatal. The victorious HENRY had scarcely reached London, when the malady burst forth, and the Lord Mayor and sixteen Aldermen died within a week. The disease generally selected the most robust and vigorous for its victims. Scarcely one in one hundred recovered at first; but it passed over the metropolis like a flash of lightning, as it were, and terminated its career in five weeks. One attack was no security against a second, or even a third. It spread over the whole country, as nearly equally fatal every where as in the metropolis.

### THE PHYSICIANS.

They could do no good in this terrific scourge, and they are nowhere alluded to in the histories of the Epidemic! Even LINACRE, who had experienced three attacks of the malady, in his own person, gives no account of it in any of his writings! The people, left to their own resources, adopted the wisest method of treatment. They avoided all violent medicines—abstained from food—applied moderate heat—and drank only a small quantity of mild fluids. On the first of January 1486, a violent tempest arose from the East, purified the atmosphere, and swept the Epidemic clear out of town and country.

### CAUSES.

This Epidemic confined itself entirely to England, and ventured not into Ireland, Scotland, or even Calais, then in our possession. "It plainly appeared, (says Hecker,) in the sequel, that the English SWEATING SICKNESS was *a Spirit of the Mist*, which hovered amid the dark clouds." Meantime other European nations, though they escaped the Sweating

Sickness, were scourged by various other epidemics, of longer duration, but not quite so fatal.

" It was (says our author) an inflammatory rheumatic fever, with great disorder of the nervous system. This assumption is supported by the manner of its origin and its especial characteristic of being accompanied by a profuse and injurious perspiration. From the judgment that we are now capable of forming of the pernicious influences which prevailed in the year 1485, it may, without hesitation, be admitted that the humidity of that and of the preceding years affected the functions of the lungs and of the skin, and disturbed the relation of this very important tissue to the internal organs of life. This is the usual commencement of rheumatic fevers, which bear the same relation to the sweating sickness as slight symptoms bear to severe ones of the same kind. The predominance of affections of the brain and of the nerves, however, gave to the English epidemic a peculiar character. The functions of the eighth pair of nerves were violently disordered in this disease, as was shewn by oppressed respiration and extreme anxiety with nausea and vomiting, symptoms to which the moderns attach much importance." 191.

It is scarcely worth while to notice this fanciful pathological hypothesis, since neither Hecker, nor any one else, can form the slightest idea of this mysterious malady. Between Sweating Sickness and Acute Rheumatism, there certainly is very slight analogy. Rigors usher in almost all fevers of an inflammatory nature, and perspiration is far from being common in the first day's rheumatic fever.

### SECOND VISITATION.

In 1506, this Epidemic again appeared. Great revolutions were now taking place in Europe. Printing was invented to dispel the clouds of darkness—a new world was discovered, to excite the energies of mankind —the circumnavigation of Africa opened golden prospects in the East—regal power was fixed on a firmer basis—standing armies were established as the pillars of civil order, but at the same time as the instruments of tyranny and oppression. It was at this time, too, that syphilis raised its mysterious head, and spread over Europe, with the rapidity of lightning. Meantime, in the Summer of 1506, the Sweating Sickness suddenly broke out in London, and, after a few weeks, again disappeared. In this second visitation, as in the first, " the malignant fever was left to the curative powers of Nature, and no powerful medicines administered." The result was extremely favourable—" in few houses did any fatal cases occur." As usual, medical histories of the Epidemic are nowhere to be found; and whether it penetrated through England, or was confined to London, does not appear. Cotemporary with this mild epidemic in England, however, there were many prevalent scourges, of different kinds, on the Continent, as the petechial fever in Italy, &c. &c.

In July, 1517, the third eruption of the Sweating Sickness occurred in London, under the mild sway of our Eighth Henry.

" On this occasion it was so violent and so rapid in its course, that it carried off those who were attacked, in two or three hours, so that the first shivering fit was regarded as the announcement of certain death. It was not ushered in by any precursory symptoms. Many who were in good health at noon, were numbered among the dead by the evening, and thus as great a dread was created at this new peril as ever was felt during the prevalence of the most suddenly destructive epidemic: for the thought of being snatched away from the full enjoyment of existence without any preparation, without any hope of recovery, is appalling even to the bravest, and excites secret trepidation and anguish. Among the lower classes the deaths were innumerable. The city was moreover crowded with poor; but even the ranks of the higher classes were thinned, and no precaution averted death from their palaces." 210.

Although very fatal, it subsided in September, and was followed by the Plague. This visitation appears to have spread through England. In Oxford and Cambridge it carried off many distinguished scholars. Scotland and Ireland were spared.

No particular atmospheric or telluric phenomena preceded this epidemic, or threw any light on its etiology. Its contagious character was, of course, believed in, but if contagion existed, it is rather strange that the disease never penetrated into Scotland or Ireland.

The fourth devastation of the Sweating Sickness in England, took place in 1528-9. But, at this time, Europe itself was scourged by the same pestilence, which began in France, after the disasters of their army before Naples, and spread in all directions. In London it broke out in the latter end of May, and spread rapidly over the whole country. The mortality of this outbreak cannot be even guessed at, though it must have been prodigious.

"As soon as the occurrences of this unfortunate year could be more closely surveyed, a conviction was at once felt, that *it was one and the same general cause of disease which called forth the poisonous pestilence in the French camp before Naples, the putrid fever among the youth in France, and the sweating sickness in England, and that the varying nature of these diseases depended only on the conditions of the soil and the qualities of the atmosphere in the countries which were visited.*" 240.

We need not follow Dr. Hecker in his account of the Sweating Sickness, which now spread over Germany, the North of Europe, &c.

"The alarm which prevailed in Germany surpasses all description, and bordered upon maniacal despair. As soon as the pestilence appeared on the continent, horrifying accounts of the unheard-of sufferings of those affected, and the certainty of their death, passed like wild-fire from mouth to mouth. Men's minds were paralysed with terror, and the imagination exaggerated the calamity, which seemed to have come upon them like a last judgment. The English Sweating Sickness was the theme of discourse everywhere, and if any one happened to be taken ill of fever, no matter of what kind, it was immediately converted into this demon, whose spectre form continually haunted the oppressed spirit. At the same time, the unfortunate delusion existed, that whoever wished to escape death when seized with the English pestilence, *must perspire for twenty-four hours without intermission.* So they put the patients, whether they had the Sweating Sickness or not, (for who had calmness enough to distinguish it?) instantly to bed, covered them with feather-beds and furs, and whilst the stove was heated to the utmost, closed the doors and windows with the greatest care to prevent all access of cool air. In order, moreover, to prevent the sufferer, should he be somewhat impatient, from throwing off his hot load, some persons in health likewise lay upon him, and thus oppressed him to such a degree, that he could neither stir hand nor foot, and, finally in this rehearsal of hell, being bathed in an agonizing sweat, gave up the ghost, when, perhaps, if his too officious relatives had manifested a little discretion, he might have been saved without difficulty." 258.

One physician, whose name has sunk in oblivion, opposed this barbarous mode of treatment, and went about from house to house, dragging the smothering patients from their beds of torture with his own hands—and thus preserving more lives than he could have done by physic.

We must now bring our notice of Hecker's work to a close. It is greatly and unnecessarily swelled out by matters very little relevant to the chief subjects. His researches into the causes of the diseases, moral, physical, religious, or political, are not merely unsatisfactory, but, in most cases, futile. Thus, in speaking of the Sweating Sickness in Germany, he endeavours to connect its etiology with the Reformation!

"The excitement was beyond all bounds. The new doctrine took root in towns and villages, but nevertheless, the most mortal party hatred raged on all sides, and, as usually happens in times of such impassioned commotion, selfishness was the animating spirit which ruled on both sides, and seized the torch of faith, in order, for her unholy purposes, to envelop the world in fire and flames." 259.

It is much more consonant with observation and experience, to find times of moral, political or religious excitement and perturbation, preventive of, rather than conducive to, epidemic diseases. It is not during the bustle of a campaign that the soldier is liable to illness, but after the hurly-burly is over.

We may remark, in conclusion, that the Epidemics of the Middle Ages, are merely matters of curiosity; and we do not believe that they do, or ever will, throw a single ray of light on the etiology, pathology, or therapeutics of epidemics in general, or of themselves in particular.

